# Morphology and Degradation of Multicompartment Microparticles Based on Semi-Crystalline Polystyrene-*block*-Polybutadiene-*block*-Poly(*L*-lactide) Triblock Terpolymers

**DOI:** 10.3390/polym13244358

**Published:** 2021-12-13

**Authors:** Nicole Janoszka, Suna Azhdari, Christian Hils, Deniz Coban, Holger Schmalz, André H. Gröschel

**Affiliations:** 1 Physical Chemistry, Center for Soft Nanoscience (SoN) and Center for Nanotechnology (CeNTech), University of Münster, Corrensstraße 28-30, 48149 Münster, Germany; nicole.janoszka@uni-muenster.de (N.J.); azhdari@exchange.wwu.de (S.A.); dcoban@exchange.wwu.de (D.C.); 2 Macromolecular Chemistry II, University of Bayreuth, Universitätsstraße 30, 95440 Bayreuth, Germany; christian.hils@basf.com; 3 Bavarian Polymer Institute (BPI), University of Bayreuth, Universitätsstraße 30, 95440 Bayreuth, Germany

**Keywords:** 3D confinement, ABC triblock terpolymers, degradation, emulsification, microparticles

## Abstract

The confinement assembly of block copolymers shows great potential regarding the formation of functional microparticles with compartmentalized structure. Although a large variety of block chemistries have already been used, less is known about microdomain degradation, which could lead to mesoporous microparticles with particularly complex morphologies for ABC triblock terpolymers. Here, we report on the formation of triblock terpolymer-based, multicompartment microparticles (MMs) and the selective degradation of domains into mesoporous microparticles. A series of polystyrene-*block*-polybutadiene-*block*-poly(*L*-lactide) (PS-*b*-PB-*b*-P*L*LA, SBL) triblock terpolymers was synthesized by a combination of anionic vinyl and ring-opening polymerization, which were transformed into microparticles through evaporation-induced confinement assembly. Despite different block compositions and the presence of a crystallizable P*L*LA block, we mainly identified hexagonally packed cylinders with a P*L*LA core and PB shell embedded in a PS matrix. Emulsions were prepared with *Shirasu Porous Glass* (SPG) membranes leading to a narrow size distribution of the microparticles and control of the average particle diameter, *d* ≈ 0.4 µm–1.8 µm. The core–shell cylinders lie parallel to the surface for particle diameters *d* < 0.5 µm and progressively more perpendicular for larger particles *d* > 0.8 µm as verified with scanning and transmission electron microscopy and particle cross-sections. Finally, the selective degradation of the P*L*LA cylinders under basic conditions resulted in mesoporous microparticles with a pronounced surface roughness.

## 1. Introduction

Block copolymers (BCPs) were demonstrated to be highly versatile materials to pattern surfaces and serve as templates for inorganic nanostructures [1,2]. They are also prime candidates for the self-assembly in solution, giving access to diverse micelle morphologies with tuneable dimensions [3,4], near-monodisperse fibres and platelets through crystallization-driven self-assembly [5,6,7], and complex nanostructures through hierarchical processes [8,9].

More recently, the microphase behaviour of BCPs in confinement has become another versatile method of forming BCP nanostructures with particular interest for the design of functional microparticles. With respect to AB diblock copolymers, a number of works clarified the effect of block composition [10], architecture [11,12], evaporation rate [13,14], type of surfactant [15,16,17] and emulsification method [18]. The focus progressively shifts towards more complex compositions (e.g., BCP nanoparticle hybrids) [19,20], as well as functionality, including response to magnetic fields, temperature, and light [21,22,23]. Another way to increase the functionality of the microparticles is the use of ABC triblock terpolymers [24,25,26,27] that feature three sequentially linked blocks with different chemistries that are known for their rich microphase behaviour [28,29,30]. Terpolymers in confinement have led to a variety of confinement-specific morphologies and nanoparticles, e.g., nanotoroids from lamella-ring morphology [31], nanocups from hemispherical lamella–lamellae [32] and perforated discs from axially stacked perforated lamellae [33]. Especially in combination with SPG membrane emulsification, the size of microparticles and nanoparticles can be controlled rather precisely.

Although a range of morphologies has been explored so far, individual microdomains or compartments have not been utilized much, regarding selective loading or removal, to create defined channel systems or porosity. The latter could, however, prove useful in the design of porous scaffolds for capture and release, catalysis, or the formation of mesoporous carbon. There are multiple, conceivable ways to hollow out individual compartments, e.g., the use of additives (homopolymer, hydrogen [34] or halogen [35,36] bonding) that can be washed out selectively or the use of degradable polymers. Among these, poly(*ε*-caprolactone) (PCL) and polylactide (PLA) are the most prominent examples, along with upcoming alternatives (e.g., polyphosphoesters) [37]. PLA and PCL and their copolymers are part of active research, where ecologically more viable catalysts are currently being identified [38,39,40], as well as degradable materials [41] and nanostructures with predetermined release profiles [42]. With respect to the confinement assembly of degradable BCPs, only a few studies exist that address polystyrene-*block*-poly(*D*-lactide) (PS-*b*-P*D*LA) BCPs [43,44,45]. There, the morphology was found to depend on the evaporation rate, solvent quality and temperature, leading to helical P*D*LA cylinders embedded in a PS matrix, axially stacked rings, or P*D*LA networks. Inspired by these works, we sought to add another polymer block/domain to the system, in order to introduce a functional surface layer after P*D*LA removal. In PS-*b*-P*D*LA microparticles, P*D*LA can be removed to form pores, while the purpose of PS is to retain the shape and structural integrity of the microparticles. Adding another block between PS and P*D*LA, e.g., polybutadiene (PB), makes it possible to further utilize the surface layer of the pore system of the microparticles for functionalization after P*D*LA removal (e.g., thiol-ene click chemistry). Therefore, the microparticles of PS-*b*-PB-*b*-P*L*LA (polystyrene-*block*-polybutadiene-*block*-poly(*L*-lactide), SBL) could provide a route toward surface-functional, porous microparticles with a versatile post-modification capability.

In this work, we lay the foundation for this direction by employing 3D confinement assembly as a structuring method to form MMs of SBL triblock terpolymers with a semi-crystalline and degradable P*L*LA microdomain. We studied the influence of block composition on inner morphology, as well as the effect of SPG membrane pore diameter on microparticle diameter and size distribution. Finally, microparticles were subjected to basic conditions to analyse the selective degradation of the P*L*LA microdomain, resulting in mesoporous microparticles.

## 2. Materials and Methods

All chemicals were used as received unless otherwise noted. Styrene (≥99.9%, ReagentPlus^®^, Sigma-Aldrich, Taufkirchen, Germany) was purified over di-*n*-butylmagnesium ((*n*-Bu)_2_Mg). 1,3-Butadiene (gaseous, Messer Industriegase GmbH, Bad Soden, Germany) was passed through columns filled with molecular sieve (4 Å) and basic aluminium oxide, condensed into a glass reactor, and stirred over (*n*-Bu)_2_Mg at least two days prior to use [46]. Ethylene oxide (3.0, Linde GmbH, Pullach, Germany) was condensed onto calcium hydride (CaH_2_) and stirred at 0 °C for 3 h before being transferred into a storage ampoule [47]. Additionally, ethylene oxide was purified over *n*-butyllithium at 0 °C directly before use, followed by condensation into a glass ampoule. *L*-lactide (≥99.8%, PURASORB^®^ L, Corbion, Amsterdam, The Netherlands) was recrystallized from toluene and stored under nitrogen until use. Dichloromethane (DCM, ≥99.5%, AnalaR NORMAPUR, VWR International GmbH, Darmstadt, Germany) used for polymerization was dried by successive distillation over CaH_2_. Triethylaluminium (Et_3_Al, 1 M in hexane, Sigma-Aldrich, Taufkirchen, Germany), (*n*-Bu)_2_Mg (0.5 M in heptane, Sigma-Aldrich), *n*-butyllithium (1.6 M in hexanes, AcroSeal^®^, Thermo Fisher Scientific, Geel, Belgium), *sec*-butyllithium (*sec*-BuLi, 1.3 M in cyclohexane/hexane (92/8), AcroSeal^®^, Thermo Fisher Scientific, Geel, Belgium), sodium dodecyl sulfate (SDS, >99%, Sigma-Aldrich, Taufkirchen, Germany), osmium tetroxide (OsO_4_, 4 wt.% in H_2_O, Science Services, Munich, Germany), trimethylsilyl chloride (TMSC, ≥99%, Sigma Aldrich, Taufkirchen, Germany), chloroform (CHCl_3_, analytical grade, Merck Chemicals GmbH, Darmstadt, Germany), sodium hydroxide (NaOH, 99.1%, VWR International GmbH, Darmstadt, Germany), and methanol (MeOH, >99.9%, Thermo Fisher Scientific, Geel, Belgium) were used as received. All polymerizations were performed under a dry argon or nitrogen atmosphere using Schlenk techniques and glass reactors for anionic polymerization to exclude moisture and air during the polymerization. Ultrapure water from a Milli-Q Integral Water Purification System was used for the preparation of emulsions and for purification.

### 2.1. Synthesis of SB-OH Precursors

The OH-terminated polystyrene-*block*-poly(1,4-butadiene) (PS-*b*-PB-OH, SB-OH) diblock copolymers were synthesized by sequential anionic polymerization in toluene initiated with *sec*-BuLi. The end-functionalization of the polymer was completed with ethylene oxide. The polymer was precipitated from MeOH, filtered, and dried in vacuo.

### 2.2. Synthesis of SBL Triblock Terpolymers

The P*L*LA blocks were synthesized by anionic ring-opening polymerization according to established recipes [48,49]. In brief, the synthesis of polystyrene-*block*-poly(1,4-butadiene)-*block*-poly(*L*-lactide) (PS-*b*-PB-*b*-P*L*LA, SBL) was realized by polymerization of *L*-lactide with DBU as a catalyst and SB-OH as macroinitiator in DCM. In the terpolymer notation employed here (AxByCz), the subscripts denote the number average degree of polymerization of the respective block (Table 1).

### 2.3. Preparation of SBL Bulk Films

Before preparation of bulk films, each vial was hydrophobized with trimethylsilyl chloride prior to use. For each bulk film, 30 mg of SBL triblock terpolymer was dissolved in 1 mL DCM and stirred for 1 h, followed by slow evaporation of DCM over 5 days. After bulk film formation, the film was removed by cooling the vial with liquid nitrogen and breaking the glass vial.

### 2.4. Preparation of SBL Microparticles

SBL triblock terpolymers were dissolved in CHCl_3_ at a concentration of *c* = 10 g L^−1^ and stirred overnight prior to emulsification. A stock solution of SDS with a concentration of *c* = 5 g L^−1^ was prepared as the continuous phase and 35 mL was used for each emulsification experiment. The emulsification process used a SPG membrane with different pore diameters of *d*_pore_ = 0.3, 0.5, 0.8, 1.1 and 2.0 µm. The 50 mL reservoir was stirred at 250 rpm while argon pressure continuously pressed the polymer solution through the membrane. After emulsification, the organic phase evaporated over three days at room temperature before the solid SBL microparticles were dialyzed against ultrapure water. The dialysis bath was exchanged 5 times to remove excess SDS.

### 2.5. Degradation of SBL Microparticles

The SBL microparticles were degraded in solution according to the literature [43]. The microparticles were dispersed in a NaOH solution (*c* = 20 g L^−1^) of H_2_O/MeOH (40/60 *v/v*) to yield a microparticle concentration of *c* = 1 g L^−1^. The particle suspension was stirred for 5 days. After degradation, the remaining MMs were dialyzed against ultrapure water to remove NaOH and impurities.

### 2.6. Nuclear Magnetic Resonance (NMR) Spectroscopy

The SBL triblock terpolymers were characterized by ^1^H-NMR spectroscopy (Bruker Ultrashield 300 spectrometer, Bruker Corporation, Billerica, MA, USA) using CDCl_3_ as solvent. The signal assignment was supported by simulations with the NMR software MestReNova. Absolute number average block lengths were determined from the ^1^H-NMR spectra, employing the number average molecular weight (*M*_n_) of the PS precursor, determined by SEC with PS calibration for signal calibration.

### 2.7. Size-Exclusion Chromatography (SEC)

SEC measurements were performed on a SEC 1260 Infinity system (Agilent Technologies, Santa Clara, CA, USA) with two PSS-SDV gel columns (particle size = 5 µm) with porosity range from 10^2^ to 10^5^ Å (PSS, Mainz, Germany). CHCl_3_ (HPLC grade) was used as eluent. All samples were dissolved and filtered through a 0.2 µm PTFE filter before analysis. The samples were measured at a flow rate of 0.5 mL min^−1^ at 23 °C, using a refractive index detector (Agilent Technologies, Santa Clara, CA, USA). The calibration was completed with narrowly distributed PS standards (PSS calibration kit), and toluene (HPLC grade) was used as internal standard.

### 2.8. Differential Scanning Calorimetry (DSC)

Calorimetric measurement of the SBL bulk films was performed on a Phoenix 204 F1 (Netzsch, Selb, Germany) instrument using aluminium crucibles (pierced lid) and nitrogen as carrier gas. The temperature range was selected from −150 °C to 200 °C (liquid N_2_ cooling) with scanning rates of 10 K min^−1^.

### 2.9. Transmission Electron Microscopy (TEM)

SBL microparticles were analysed on a JEOL JEM-1400 Plus TEM operating at an acceleration voltage of 120 kV. The diluted particle dispersion (*c* = 0.3 g L^−1^) was dropped on a carbon-coated copper grid. The sample was blotted with a filter paper after 30 s and the grid was dried for at least 12 h. The samples were stained with OsO_4_ for 1 h before analysis. The TEM images were processed with ImageJ (version 1.53c).

### 2.10. Scanning Electron Microscopy (SEM)

The surface and shape of the microparticles were recorded on a cryo-field emission Zeiss Crossbeam 540 FIB-SEM equipped with lens-, chamber-, and energy-selective detectors for 16 Bit image series acquisition with up to a 40,000 × 50,000-pixel resolution. Samples were prepared by dropping 20 µL of dispersion with a concentration of *c* = 0.3 g L^−1^ on a silicon wafer. The solution was blotted with a filter paper after 30 s and the wafer was dried for at least 12 h. Before recording images, a Pt-Cd layer of 6 nm was sputtered onto the sample using a Quorum PP3010T-Cryo chamber with integrated Q150T-Es high-end sputter coater and Pt–Cd target.

### 2.11. Ultra-Sectioning of SBL Microparticles

Freeze-dried SBL microparticles were embedded in 3D Rapid Resin CLEAR 3DR3582C, cured by UV light (λ = 365 nm), and sectioned with a Reichert/Leica Ultracut E microtome (Leica Microsystems, Wetzlar, Germany). A section velocity of around 1 mm s^−1^ with an inclination angle of 6 degrees was used to gain cross-sectional slices of about 70 nm thickness. For TEM imaging, cross-sections were transferred on a carbon-coated copper grid. The PB domains were selectively stained with OsO_4_ for 3 h and followed by sputtering the samples with a carbon coating of 10 nm before imaging.

### 2.12. Raman Spectroscopy

The Raman spectra of the SBL-56 microparticles before and after hydrolytic degradation of the P*L*LA block were taken with a WITec alpha 300 RA+ imaging system (WITec GmbH, Ulm, Germany), equipped with an UHTS 300 spectrometer and a back-illuminated Andor Newton 970 EMCCD (electron multiplying charge-coupled device) camera. For the measurements an excitation wavelength of λ = 532 nm and a 50× objective (Zeiss LD EC Epiplan-Neofluar, NA = 0.55) together with the WITec suite FIVE 5.3 software package were used. Spectra were acquired using a laser power of 10 mW, an integration time of 0.5 s and 50 accumulations. All spectra were subjected to a cosmic ray removal routine and base line correction. Samples were prepared by placing some drops of the dispersions onto glass slides followed by drying.

### 2.13. Dynamic Light Scattering (DLS)

SBL microparticles were measured on a LSI spectrometer (Fribourg, Switzerland) operating with a DPSS Cobolt laser (max. 100 mW constant output at λ = 660 nm). Samples were prepared at a concentration of *c* = 0.06 g L^−1^ and purified three times from dust by passing the sample solution through a PTFE filter with a pore size of 5 µm directly into cylindrical quartz cuvettes (d = 10 mm), which were flushed with compressed air before measurement. Three intensity–time autocorrelation functions were measured at a scattering angle of 90° and an acquisition time of 60 s. Recorded data were analysed with the LSI software package (v.63).

## 3. Results and Discussion

**Scheme 1:**
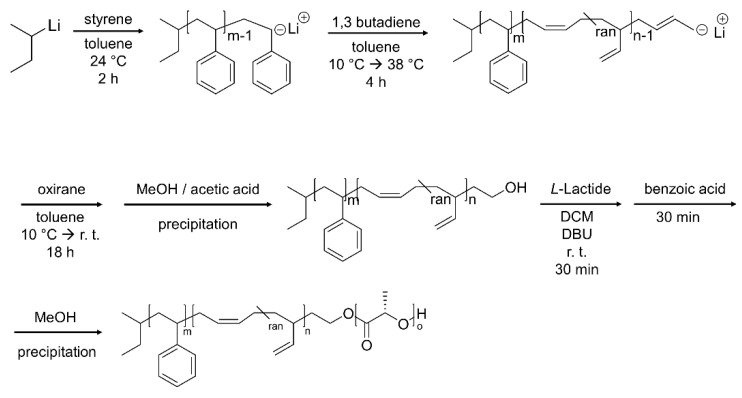
**Synthesis of SBL triblock terpolymers.** Sequential anionic polymerization of styrene and 1,3-butadiene, and termination with oxirane leads to OH-modified polystyrene-*block*-poly(1,4-butadiene) (SB-OH). Anionic ring-opening polymerization of *L*-lactide afforded the SBL triblock terpolymers.

We first synthesized SBL triblock terpolymers with different weight fractions of the poly(*L*-lactide) (P*L*LA) block ($f_{L}$ = 0.1–0.56) to prepare MMs with different morphologies. For the SBL syntheses, a two-step procedure was employed as summarized in Scheme 1. First, a hydroxy-terminated polystyrene-*block*-poly(1,4-butadiene) (SB-OH) precursor was prepared by sequential living anionic polymerization of styrene and 1,3-butadiene in toluene, followed by quantitative end capping with ethylene oxide. Subsequently, SB-OH was used as macroinitiator for the anionic ring-opening polymerization of *L*-lactide in DCM in the presence of DBU as catalyst. This yielded a series of SBL triblock terpolymers (Table 1), SBL-10, SBL-37, and SBL-52, as well as SBL-56, synthesized from two different SB-OH precursors (S_118_B_310_-OH and S_295_B_292_-OH). In the used SBL-X notation, X stands for the weight fraction of the P*L*LA block.

**Figure 1:**
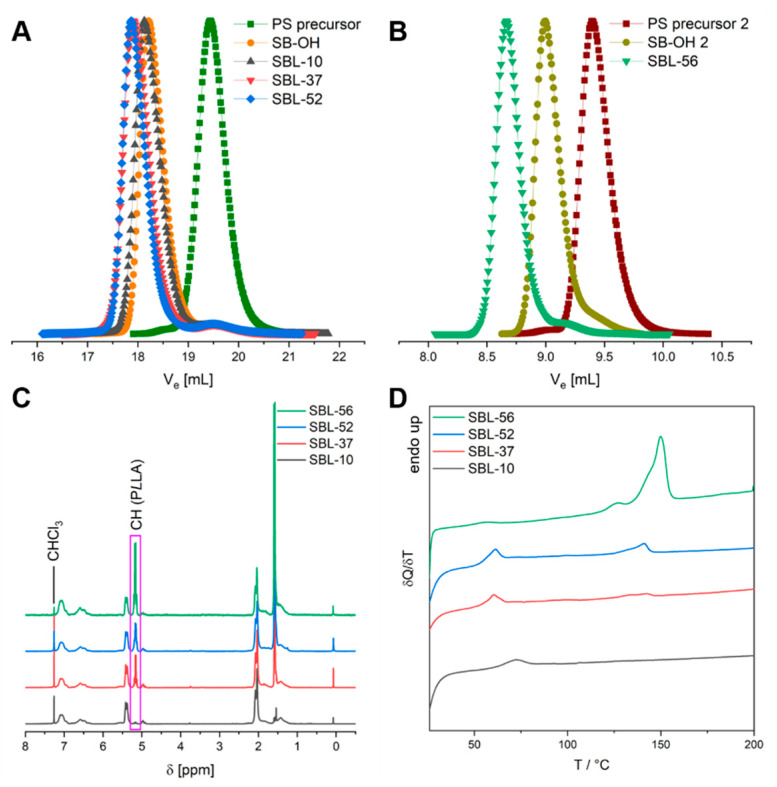
**Characterization of the SBL triblock terpolymers.** (**A**,**B**) SEC traces for the two series of terpolymers based on S_118_B_310_-OH and S_295_B_292_-OH macroinitiators and the respective PS precursors. (**C**) ^1^H-NMR spectra of all SBLs with characteristic P*L*LA peak being highlighted and (**D**) DSC first heating traces of bulk films cast from DCM.

According to SEC, all synthetic steps proceeded without significant side reactions, resulting in narrowly distributed SBLs with dispersities of *Đ* ≈ 1.1–1.2 (Figure 1A,B and Table 1). In the ^1^H-NMR spectra, the signal at 5.2 ppm can be assigned to the methine protons of the P*L*LA repeating unit, showing an increasing peak area in the series SBL-10 to SBL-56 (Figure 1C). The final composition of the SBL triblock terpolymers was calculated by comparing the signals of PS, PB and P*L*LA, whereas the molecular weight of PS obtained from SEC served as a reference. In order to probe whether the P*L*LA block is able to crystallize, differential scanning calorimetry (DSC) was performed on SBL bulk films, which were cast from DCM (Figure 1D). Here, only the first heating traces, i.e., the thermal properties directly after film casting, were considered, as these came closest to the thermal properties of the SBL microparticles. For SBL-10, no melting endotherm could be observed for the P*L*LA block, most likely due to its low volume fraction. In addition, SBL-10 showed only one glass transition temperature ($T_{g}$), being located in between the expected $T_{g}$-values of PS ($T_{g}$ = 100 °C) and P*L*LA ($T_{g}$ = 50 °C), pointing to the formation of a mixed amorphous PS/P*L*LA phase. For SBL-37, SBL-52, and SBL-56 melting endotherms confirmed that a partial crystallization of P*L*LA occurs upon film casting with increasing P*L*LA content. This effect is most pronounced for SBL-56, which has the highest P*L*LA fraction as well as highest overall molecular weight.

**Scheme 2:**
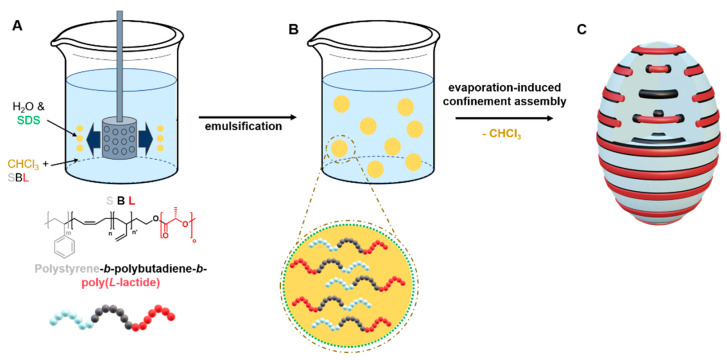
**Preparation of the SBL MMs through SPG membrane emulsification and evaporation-induced confinement assembly.** (**A**) Emulsification of SBL with the SPG membrane. (**B**) Microemulsion droplets of SBL/CHCl_3_ in aqueous solution stabilized by SDS. (**C**) Resulting MMs after solvent evaporation process.

After SBL synthesis, we obtained MMs through SPG membrane emulsification followed by the evaporation of the organic solvent (evaporation-induced confinement assembly (EICA)). Scheme 2 outlines the process of obtaining SBL MMs. The SPG method utilizes porous glass membranes through which the polymer solution is pressed into the continuous surfactant phase to generate emulsion droplets of a homogeneous size. For that, SBL was first dissolved in CHCl_3_ at a concentration of $c_{\mathrm{SBL}}=10\;{g\;L}^{-1}$ and stirred overnight prior to emulsification. To form the oil-in-water (O/W) emulsion, the SBL solution was pushed through the SPG membrane with argon pressure into the continuous phase consisting of an aqueous sodium dodecyl sulfate solution (SDS, $c_{\mathrm{SDS}}=5\;{g\;L}^{-1}$). Depending on the pore size of the membrane, the SBL solution was completely emulsified within 2–3 h and CHCl_3_ was evaporated over three days under an ambient atmosphere while gently stirring the emulsion. We utilized five different membrane pore sizes (*d*_pore_ = 0.3, 0.5, 0.8, 1.1, and 2.0 µm) to investigate the size distribution and morphology of each of the SBL MMs.

**Figure 2:**
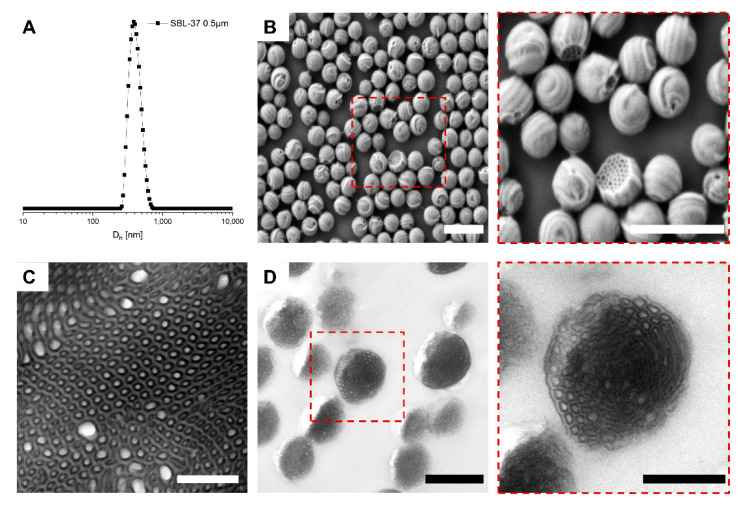
**SBL-37 MMs prepared with the membrane *d_pore_* = 0.5 µm.** (**A**) Size distribution from DLS CONTIN plot. (**B**) SEM overview image and close-up (scale bars 1000 nm). (**C**) TEM image of the SBL-37 bulk morphology (scale bar 500 nm) and (**D**) TEM of cross-sections of embedded SBL-37 MMs (*d*_pore_ = 2.0 µm) visualizing the inner morphology (TEM samples stained with OsO_4_ to enhance the contrast of PB; P*L*LA appears brightest, scale bars 1000 nm and 500 nm in inset).

We started our analytics by first verifying the suitability of the SPG method to form MMs from SBL triblock terpolymers. Figure 2 summarizes the results of SBL-37 MMs fabricated with a pore diameter of *d*_pore_ = 0.5 µm. DLS measurements suggest a narrow size distribution of the SBL-37 particles, as the CONTIN analysis shows a narrow fit with an average hydrodynamic diameter of *D*_h_ = 490 nm (*Đ* = 0.15) (Figure 2A). According to SEM analysis (Figure 2B), the SBL-37 MMs exhibited an average particle diameter of *d* = 388 ± 48 nm and were frequently arranged in a hexagonal lattice upon drying on the silicon wafer corroborating the narrow size distribution realized with the SPG method. The *D*_h_ of the SBL-37 MMs is slightly larger than the diameter in SEM, which we attribute to shrinking due to *e*-beam damage (mostly degrading the P*L*LA block). Nevertheless, the *D*_h_ fits with the expected particle size produced with a 0.5 µm pore diameter of the SGP membrane.

SEM further revealed a striped surface of the SBL-37 MMs (Figure 2B) indicating a lamella or cylinder morphology. At weight fractions of $f_{S}=0.34$, $f_{B}=0.29$, and $f_{L}=0.37$, a lamella–lamella morphology is more likely, but the close-up SEM image of Figure 2B clearly shows that some particles have a flat side revealing hexagonally packed cylinders. Although it is surprising to find a core–shell cylinder morphology with a P*L*LA core and PS shell at such a large $f_{L}=37$, the corresponding TEM image of the bulk morphology corroborates this morphology (Figure 2C). Before TEM imaging, the sample was stained with OsO_4_ to enhance the contrast of the PB microdomains, which appear dark (PS light grey, P*L*LA bright). As we will see later, the core–shell cylinder morphology is stable even at $f_{L}>0.37$. Due to the anisotropy of the P*L*LA cylinder phase, the particles were not able to fully close into spheres in some cases, and therefore developed a flat side with a hexagonal perforation. The otherwise striped ’baseball‘ surface pattern is a typical defect pattern that cylinders develop in or on spherical surfaces [50,51,52,53,54]. In order to gain information about the internal structure of the microparticles, ultrathin cross-sections were generated from the largest *d*_pore_ = 2.0 µm (Figure 2D). The close-up TEM image of a single SBL-37 particle in Figure 2D verifies the core–shell cylinder morphology and their hexagonal packing. The cylinders do not appear to have a fully circular cross-sections, but instead show rectangular features. Such a deformation was observed before for core–shell cylinders of polystyrene-*block*-polybutadiene-*block*-poly(*ε*-caprolactone) (SBC) triblock terpolymers with a semi-crystalline PCL core block, but with a lower $f_{C}=0.16$ [55,56]. We therefore attribute the deviation from the spherical cross-section of the cylinders to the semi-crystalline nature of P*L*LA. If the SBL core–shell cylinders undergo one complete revolution along the curved microparticle interface, they close into core–shell rings (or toroids), an idea that was also previously reported for PS-*b*-PB-*b*-P*t*BMA (P*t*BMA = poly(*tert*-butyl methacrylate) [26].

**Figure 3:**
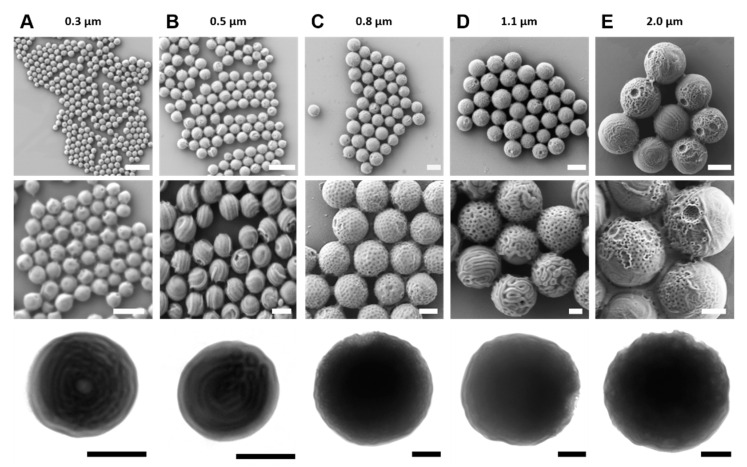
**SBL-37 MMs prepared with different *d*_pore_:** (**A**) 0.3 µm, (**B**) 0.5 µm, (**C**) 0.8 µm, (**D**) 1.1 µm, and (**E**) 2.0 µm. Top row: SEM overview images (scale bars 1000 nm); middle row: close-up SEM images (scale bars 500 nm); bottom row: TEM images (scale bars 200 nm).

To investigate the effect of membrane pore diameter on the inner morphology, we emulsified SBL-37 with five different *d*_pore_ = 0.3, 0.5, 0.8, 1.1, and 2.0 µm, and analysed the size and inner structure with TEM, SEM and DLS (Figure 3 and Figure S1). The SEM overview images exhibit a narrow size distribution for each sample implying a good control over the process (Figure 3A). The DLS measurements in Figure S1 confirm a monomodal and narrow size distribution for all SBL-37 MMs and an increasing *D*_h_ with pore size. As expected, the average diameter of the particles increases with the pore diameter from about 390 nm to 1800 nm. The cylinder morphology remains for all SBL-37 MMs, which is not that clear from the TEM images but more visible in SEM due to the characteristic surface pattern. For smaller particles (e.g., *d*_pore_ = 0.5 µm, Figure 3B middle row) the cylinders preferentially lie parallel to the particle surface (striped baseball pattern). In contrast, core–shell cylinders are able to orientate perpendicular to the particle surface for larger particles, leading to a hexagonal pattern and perforations (e.g., *d*_pore_ > 0.8 µm, Figure 3C–E, middle row). TEM images of all SBL-37 MMs were recorded to determine the internal structure and detect any size-dependent changes in the microphases (Figure 3B). The internal structure for the smallest SBL-37 MMs (*d*_pore_ = 0.3 and 0.5 µm) is visible in transmission (larger particles are rather dark). With decreasing particle diameter, the SBL-37 triblock terpolymer is more confined during emulsification. While the size of the microdomains remains unchanged, the smaller diameter means a stronger curvature, and thus higher degree of confinement, *D*/*L*_0_. Each block is therefore forced to adapt to the curvature resulting in less-ordered structures. Conversely, microphase separation in larger particles approaches thermodynamic equilibrium and results in a more-ordered core–shell cylinder morphology (close to the bulk case). Nevertheless, all SBL-37 MMs exhibit a core–shell cylindrical morphology, suggesting that the pore diameter has little effect on the morphology itself, but rather on the orientation of the morphology.

**Figure 4:**
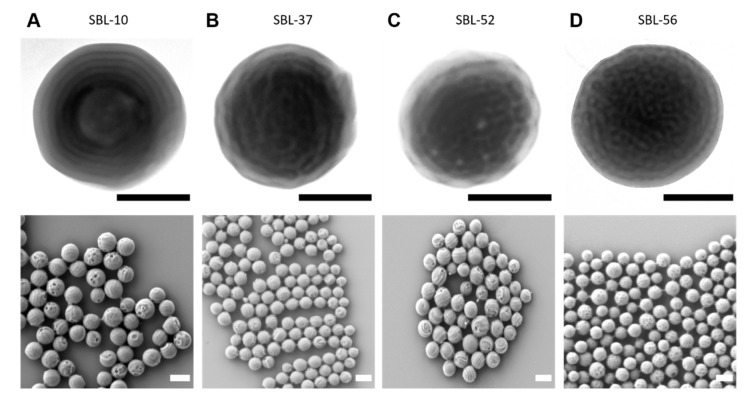
**EICA of all SBLs (as indicated) employing a membrane with *d*_pore_ = 0.3 µm.** (**A**) SBL-10, (**B**) SBL-37, (**C**) SBL-52, and (**D**) SBL-56. Top row: TEM images of individual MMs (scale bars 200 nm); bottom row: SEM overview images (scale bars 500 nm).

Next, we explored the shape and internal structure of all other SBLs. In this case, the $f_{L}$ varied from $f_{L}$ = 0.10 to $f_{L}$ = 0.56. Since the inner order of the SBL MMs is best visible for smaller particle sizes, Figure 4 summarizes MMs fabricated with a *d*_pore_ = 0.5 µm. According to TEM, an onion-like morphology is obtained for SBL-10 (Figure 4A). SBL-10 exhibits the lowest $f_{L}$, and the P*L*LA microdomain does not seem to have any impact on the final morphology, most likely due to the mixing of PS and P*L*LA chains (supported by only one *T*_g_ in DSC, see Figure 1D). Since $f_{S}$ ≈ $f_{B}$, SBL-10 is arranged in an onion-like (concentric lamellar) structure of alternating PS and PB lamellae in which P*L*LA might even be mixed with PS. Both SBL-37 and SBL-52 MMs clearly form a cylinder morphology, although the $f_{L}$ of both SBLs is quite different from each other (Figure 4B,C). Since SBL-37 with $f_{L}$ = 0.37 already formed a core–shell cylinder morphology, as discussed in Figure 2, this morphology appears to have a rather large stability region (it is also in agreement with the bulk morphology). The main difference is the core diameter of the P*L*LA microphase, which increased from *d*_cyl_ = 13 nm for SBL-37 to *d*_cyl_ = 15 nm for SBL-52. Finally, SBL-56 has the largest $f_{L}$ and exhibited a core–shell cylinder morphology, yet, was arranged as hexagonally packed and axially stacked hoops (Figure 4D). The PS still constitutes the matrix of the MMs, even though, with $f_{S}$ = 0.29, it constitutes the minority phase.

**Figure 5:**
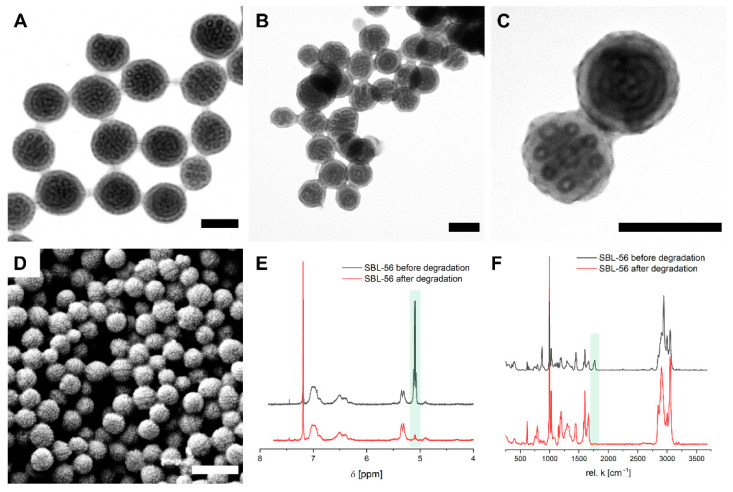
**Degradation of SBL-56 MMs under basic conditions for 5 days (membrane with *d*_pore_ = 0.3 µm).** (**A**) TEM overview image before and after degradation (**B**) (scale bars 200 nm). (**C**) TEM close-up and (**D**) SEM image after degradation (scale bars 500 nm). (**E**) ^1^H-NMR and (**F**) Raman measurements before (black) and after degradation (red).

Finally, the hydrolysis of the SBL MMs prepared with *d*_pore_ = 0.3 µm was studied to selectively remove the P*L*LA domains, and thus fabricate porous microparticles. Starting with SBL-56, which had the highest P*L*LA fraction, hydrolysis was performed for 5 days under basic conditions by stirring the SBL-56 MMs in a H_2_O/MeOH mixture (60/40 *v/v*) containing 20 g L^−1^ NaOH. We first analysed the morphology in TEM before (Figure 5A) and after degradation (Figure 5B,C). Before degradation, the MMs exhibited a core–shell cylinder structure looping into core–shell rings. As visible from the TEM images in Figure 5B,C, the ring structure remains after the hydrolysis of the P*L*LA domains. Note that the PB microphase is still present (dark rings in Figure 5C) and now covers the surface of the inner pores as well as the MM’s outer surface. The PB provides double bonds for crosslinking, loading, and versatile post-modification possibilities (e.g., through thiol-ene click reactions). Furthermore, the microparticles now exhibit a rough surface pattern in SEM (Figure 5D). The surface structure is different than before degradation (see Figure 4D) indicating the successful removal of the P*L*LA microphase. To quantify P*L*LA hydrolysis, we performed ^1^H-NMR and Raman measurements before and after degradation. The ^1^H-NMR measurements corroborate a degradation of about 95% of the P*L*LA microdomain of SBL-56 (Figure 5E). We determined the degradation by comparing the resonance peaks of the methine group of P*L*LA at about 5.2 ppm relative to the aromatic signals of PS between 6.2–7.2 ppm before and after degradation. In addition, Raman measurements also confirm the removal of the PLLA phase after 5 days by the disappearance of the characteristic carbonyl stretching vibration at ῦ = 1765 cm^−1^ (Figure 5F). We furthermore tested the basic degradation of other SBL MMs under identical conditions (Figures S2–S4). For SBL-10 MMs, the concentric lamellar structure remains after 5 days of degradation. However, in some cases, the outermost lamella of the SBL-10 MMs detached from the particle, suggesting the degradation of a mixed PS and P*L*LA phase (Figure S2). According to the TEM images displayed in Figure S3, the initial core–shell cylinder morphology of SBL-37 MMs is retained upon hydrolysis, but the MMs appear porous after basic degradation of the P*L*LA microdomain. In particular, the close-up TEM image in Figure S3C and the SEM image in Figure S3D highlight the porous structure. A similar trend was also observed for SBL-52 MMs (Figure S4). The porosity of the hydrolysed SBL-37 and SBL-52 MMs further verified the formation of a P*L*LA cylinder core and agree with the morphology assignment of the SBLs after the confinement assembly discussed above.

## 4. Conclusions

In summary, SBL triblock terpolymers with varying P*L*LA block length were synthesized and self-assembled in confinement, employing SPG membranes with different pore diameters to fabricate SBL MMs with a defined size and narrow size distribution. Despite different block compositions, this process mostly led to hexagonally packed core–shell cylinders consisting of a P*L*LA core, PB shell and a PS matrix. The increasing $f_{L}$ did not have a noticeable effect on the inner structure of the MMs, which coincided with the bulk morphology. However, the average MM diameter did influence the morphology, i.e., for particle diameters *d* < 0.3 µm, the core–shell cylinders were less ordered; while for particles with 0.3 µm < *d* < 0.8 µm, the core–shell cylinders were oriented parallel to the particle surface, and preferred a perpendicular orientation of *d* > 0.8 µm. Selective degradation of the P*L*LA cylinders under basic conditions led to pronounced surface corrugations, as well as pores that were shaped according to the preceding P*L*LA cylinders. The degradation process did not affect MM structure or pore morphology. The PB microdomain became the surface coating of the pores and the overall surface. The confinement assembly of degradable triblock terpolymers, therefore, not only allows the formation of narrowly size-distributed microparticles with a controlled inner structure, but likewise provides functional microparticles after degradation.

## Figures and Tables

**Table 1:** Composition of used SBL triblock terpolymers.

Code	*P_n_ ^a^*	*f_S_ ^b^*	*f_B_*	*f_L_*	*M_n_ ^c^*	*Đ ^d^*
SBL-10	S_118_B_310_*L*LA_27_	0.49	0.41	0.10	40,100	1.12
SBL-37	S_118_B_310_*L*LA_150_	0.34	0.29	0.37	57,900	1.14
SBL-52	S_118_B_310_*L*LA_280_	0.26	0.22	0.52	76,000	1.17
SBL-56	S_295_B_292_*L*LA_408_	0.29	0.15	0.56	105,300	1.08

*^a^ P_n_* is the number average degree of polymerization and *^b^ f_n_* the weight fraction of each block. *^c^* Number average molecular weight determined through a combination of SEC and ^1^H-NMR. *^d^* Dispersity values determined with SEC (see Section 2.7).

## Data Availability

The data presented in this study are available on request from the corresponding author.

## Supplementary Materials

The following are available online at https://www.mdpi.com/article/10.3390/polym13244358/s1, Figure S1: CONTIN plots of SBL-37 MMs; Figures S2–S4: SBL-10, SBL-37, and SBL-52 MMs prepared with a membrane pore diameter of 0.3 μm before and after degradation.
