# Supplementary material for: Morphology and Degradation of Multicompartment Microparticles Based on Semi-Crystalline Polystyrene-block-Polybutadiene-block-Poly(L-lactide) Triblock Terpolymers
Source: Polymers (Basel). 2021 Dec 13;13(24):4358. doi: 10.3390/polym13244358 (PMC8706259; doi:10.3390/polym13244358)
Supplement: Supplementary file 1 [file polymers-13-04358-s001.zip › polymers-1501446-supplementary.pdf]

Supplementary Information

to

Morphology and degradation of multicompartment microparticles based on semi-crystalline polystyrene-*block*-polybutadiene-*block*-poly(*L*-lactide) triblock terpolymers

Nicole Janoszka, Suna Azhdari, Christian Hils, Deniz Coban, Holger Schmalz, André H. Gröschel

Supporting Figures

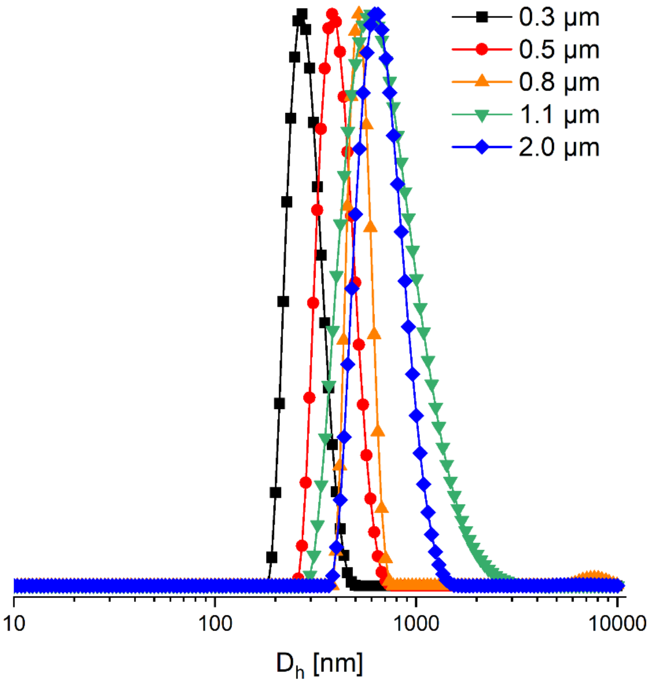

Figure S1: CONTIN plots of SBL-37 MMs prepared with different membrane pore diameter (as indicated).

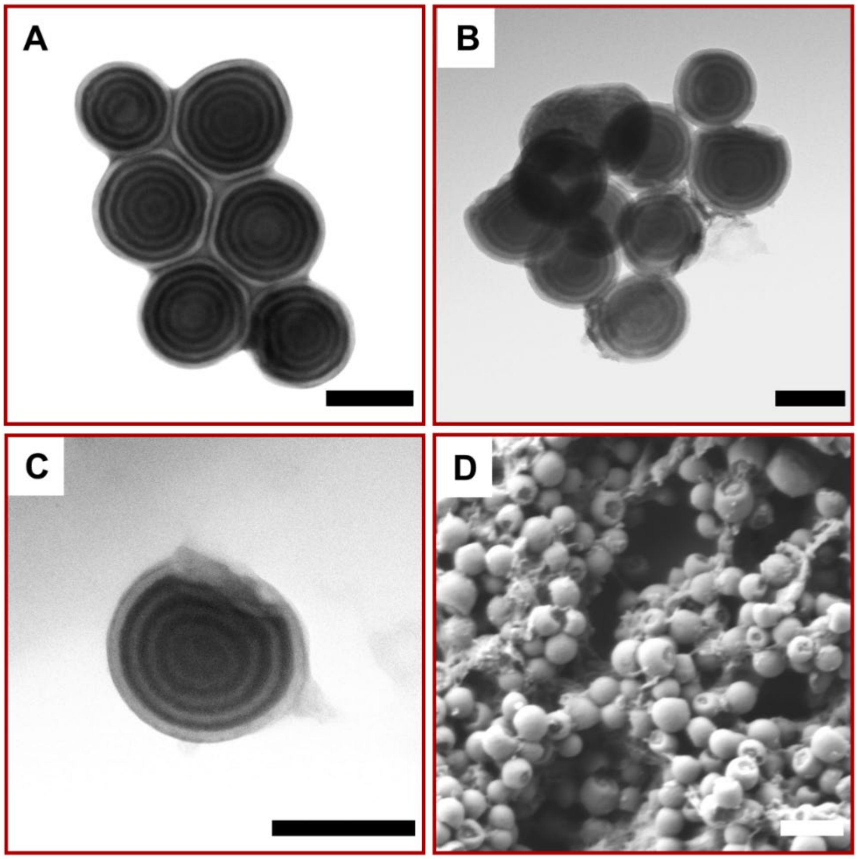

Figure S2: SBL-10 MMs prepared with a membrane pore diameter of 0.3 µm before and after degradation. A) TEM image before, **B,C)** TEM images and **D)** SEM overview image after degradation under basic conditions. Scale bars: A-C) 200 nm, D) 500 nm.

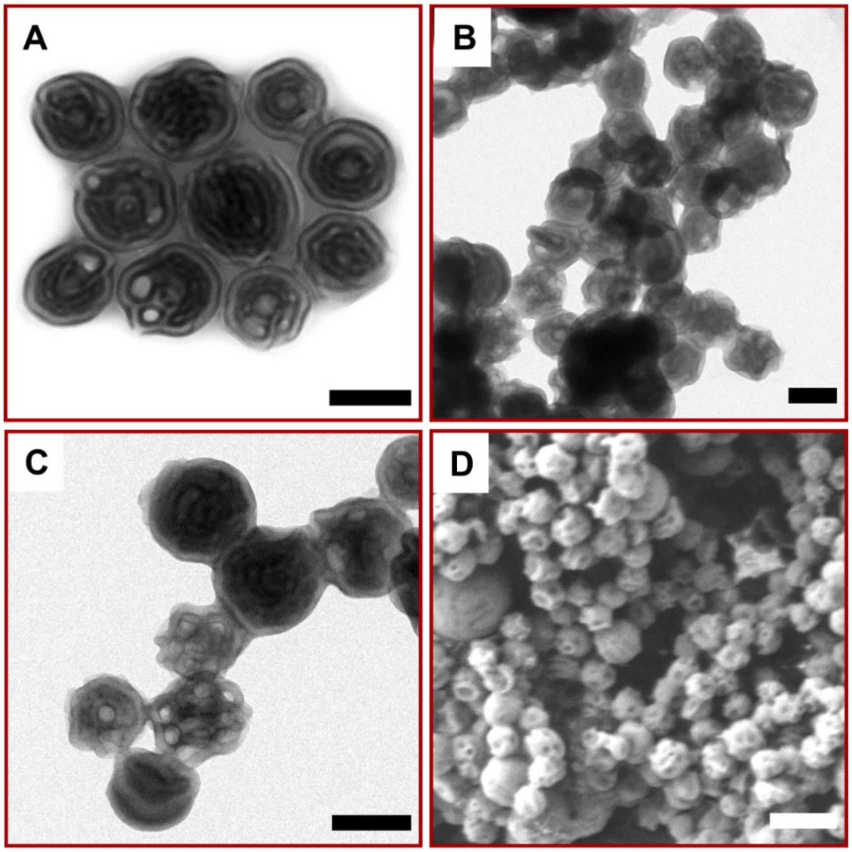

Figure S3: SBL-37 MMs prepared with a membrane size of 0.3 µm before and after degradation. A) TEM image before, **B,C)** TEM images and **D)** SEM overview image after degradation under basic conditions. Scale bars: A-C) 200 nm, D) 500 nm.

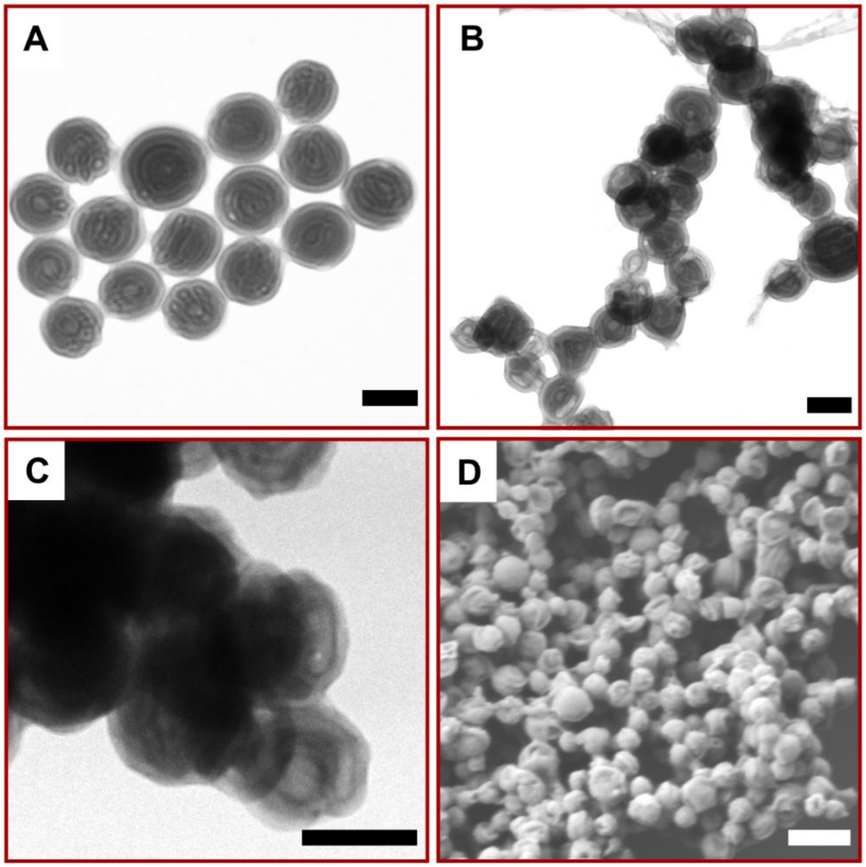

Figure S4: SBL-52 MMs prepared with a membrane size of 0.3 µm before and after degradation. **A)** TEM image before, **B,C)** TEM images and **D)** SEM overview image after degradation under basic conditions. Scale bars A-C) 200 nm, D) 500 nm.
